# Supplementary material for: Asymmetric Synthesis of Quaternary Hydantoins via a Palladium-Catalyzed Aza-Heck Cyclization
Source: J Am Chem Soc. 2025 Nov 14;147(49):44692–8. doi: 10.1021/jacs.5c16022 (PMC12703750; doi:10.1021/jacs.5c16022)
Supplement: Supplementary file 2 [file ja5c16022_si_002.zip › All NMR FID Files/S12/S12_CarbonNMR/1/pdata/1/email_MLM03071-1B-CAPT_1_1.pdf]

C13APT1024 CDCl3 /opt/nmrdata mlmurr 13

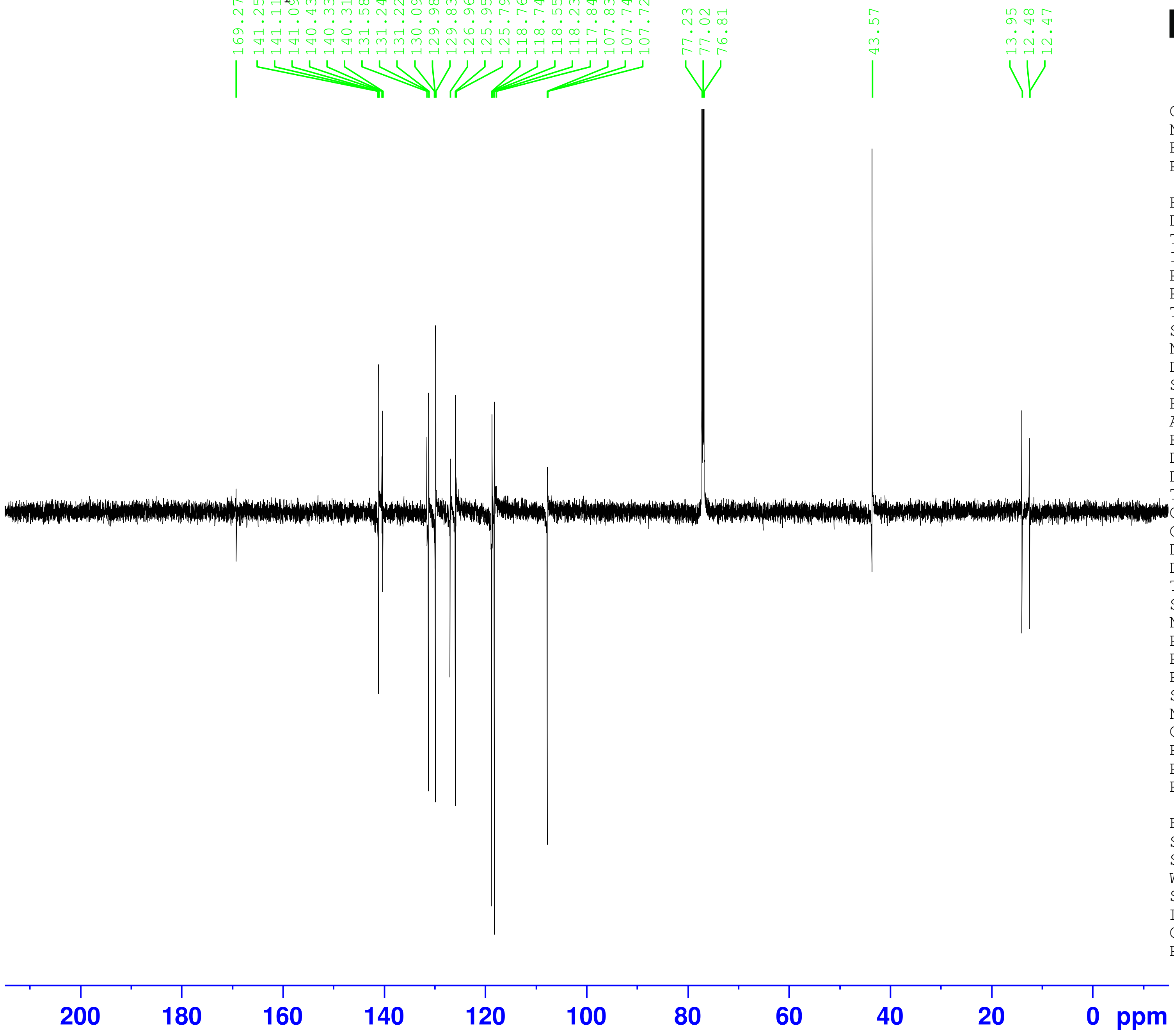

BRUKER

Current Data Parameters
NAME MLM03071-1B-CAPT
EXPNO 1
PROCNO 1

F2 - Acquisition Parameters
Date_ 20240615
Time 14.37 h
INSTRUM spect
PROBHD Z168773_0053 (
PULPROG jmod.acqt0
TD 65536
SOLVENT CDCl3
NS 1024
DS 4
SWH 34722.223 Hz
FIDRES 1.059638 Hz
AQ 0.9437184 sec
RG 1030
DW 14.400 usec
DE 18.88 usec
TE 298.0 K
CNST2 145.0000000
CNST11 1.0000000
D1 5.00000000 sec
D20 0.00689655 sec
TD0 1
SFO1 150.9656791 MHz
NUC1 13C
P1 10.00 usec
P2 20.00 usec
PLW1 88.38300323 W
SFO2 600.3224013 MHz
NUC2 1H
CPDPRG[2 waltz16
PCPD2 80.00 usec
PLW2 21.85499954 W
PLW12 0.48474309 W

F2 - Processing parameters
SI 32768
SF 150.9505840 MHz
WDW EM
SSB 0
LB 1.00 Hz
GB 0
PC 1.40
